# Experience of loneliness on well-being among young individuals: A systematic scoping review

**DOI:** 10.1007/s12144-023-04445-z

**Published:** 2023-03-03

**Authors:** Nuryn Aqidah Binte Mohammad Adib, Jagdeep Kaur Sabharwal

**Affiliations:** School of Social and Health Sciences, James Cook University, 149 Sims Drive, Singapore, 533884 Singapore

**Keywords:** Loneliness, Well-being, Young individuals, Coping, Systematic scoping review

## Abstract

A systematic scoping review was conducted to explore the current evidence on the experience of loneliness influencing well-being among youths. The electronic databases Scopus, APA PsycINFO, Emerald Insight and One Search were used to identify relevant studies, followed by an analysis of the text words contained in the title and abstract, and of the index terms used to describe the article. Reference lists of all shortlisted articles were searched for additional studies. 20 studies (quantitative, qualitative and mixed) published in the English language were identified for inclusion. Findings illustrate that the experience of loneliness is a complex, evolutionary process influenced by relational and environmental factors. Results from the studies identified factors that promote lower experience of loneliness and better well-being in future life stages. Future research can substantiate the issues related to young individuals being socially isolated from others for a prolonged duration.

Many researchers have focused on Loneliness due to its negative impacts on an individual’s physical and mental health (Cole et al., 2015; Hawkley & Capitanio, 2015; Holt-Lunstad et al., 2015) across the lifespan (Hawkley & Capitanio, 2015) and ethnicities (Alcaraz et al., 2019). Loneliness is defined as the subjective feeling of being socially isolated due to a perceived discrepancy between one’s quality and/or quantity of social relationships (Cacioppo & Patrick, 2008). According to a national survey by Cigna (2018), 46% of the sampled 20,000 American adults aged 18 years and older reported feeling lonely, with the younger generation scoring the highest on loneliness. 43% of the same sampled adults were also found to sometimes or always feel socially isolated from others. With the current COVID-19 situation, the prevalence of loneliness may increase as a result of limited social interactions with others, which if not managed, may be detrimental to physical and mental health. As such, a rise in the prevalence of loneliness may potentially lead to a decrease in well-being, which, in turn, can impose heavy societal burdens. With that being said, well-being has also been a central research focus. Transformative service research (TSR) is one of the research domains that looks towards making improvements in the well-being of individuals and their surrounding environment (Mollenkopf et al., 2020). However, the pandemic has placed tremendous pressure on individuals aged 18 to 64 years old to maintain their well-being while being socially isolated from the rest of the world (Kirzinger et al., 2020).

## Gaps in current literature

A systematic overview by Leigh-Hunt et al. (2017) noted that many have attempted to consolidate works surrounding loneliness and well-being. Most syntheses accentuate the measurement of loneliness as an indicator for well-being, as well as the development and effects of loneliness among the older individuals (e.g., Courtin & Knapp, 2017; Hawkley & Cacioppo, 2010; Stocker et al., 2020). Regardless, only a few have focused exclusively on the factors that drive loneliness in adolescents and young adults (Achterbergh et al., 2020; Korzhina et al., 2022). The reviews also fail to explicate these factors in the context of parent-child relationships and interactions with friends. Put differently, limited works have documented the experience of loneliness among young people based on their early life experiences, and how this may have impacted their current well-being. Thus, mental health practitioners, educators, and even the general public may lack ample, evidence-based insights to deal with the loneliness epidemic among younger individuals especially so during the present times.

There are some realities that urge greater attention to the experience of loneliness among adolescents and young adults. Firstly, although loneliness can affect an individual at any point in life, recent statistics from around the world report individuals aged between 16 to 25 years feeling higher intensities and more frequent feelings of loneliness (Cigna, 2018; Department for Digital, Culture Media & Sport, 2020; Weinberg & Tomyn, 2015). Secondly, very few studies have been conducted to study loneliness among the younger people. Evidence on the risk factors of loneliness and poor well-being that cluster in childhood and adolescence remain scarce. With the adolescence phase being the most risky in terms of the emergence of various mental health problems, experiencing loneliness may be stigmatising due to the adolescent’s strong need to feel connected to others (Pitman et al., 2018). This paper proposes that a deeper insight into the experience of loneliness among young people is needed to better direct prevention and treatment approaches tailored to their needs. This will be done by synthesising evidence on the effects of loneliness on well-being and understanding how the two factors are correlated.

## Review question

To manage the rising prevalence of loneliness, Pitman et al. (2018) have advised investment into raising public awareness regarding loneliness among the younger individuals since much has already been done for the older population. Whilst such investments should include increasing support from mental health services, more effort in the early detection of at-risk individuals and the promotion of concomitant loneliness prevention policies and/or practices are needed. In response, the current paper utilised a systematic scoping review methodology to synthesise evidence pertaining to the impact of loneliness on well-being. Another related purpose was to give considerations to the review’s results in providing a deeper understanding of the pathways from loneliness to well-being, and their potential to direct mental health practices. Two questions were derived from the above, which directed focus of the current review: (1) What factors contribute to experience of loneliness among young people? (2) Do existing works help us to construct a pathway between loneliness and well-being among young people?

## Systematic scoping review

Scoping reviews broadly summarise current research findings and identify any gaps within a given research topic (Arksey & O'Malley, 2005). Such reviews are particularly useful when researchers lack the time and resources to undertake a more rigorous systematic review (Arksey & O'Malley, 2005). Systematic reviews on the other hand are narrowly focused on finding answers by identifying, evaluating and synthesising all relevant studies on a particular topic (Agarwal et al., 2019; Arksey & O'Malley, 2005). The present paper merges the two procedures to find answers to specific questions from a broad body of peer reviewed literature. This paper follows the five components of the six-step approach outlined by Arksey and O'Malley (2005) to scope, identify and elaborate on the key concepts found in the extant literature regarding the association between loneliness and well-being. The steps include (a) identifying the research topic, (b) finding relevant studies, (c) selecting a study, (d) synthesising and interpreting qualitative data and (e) synthesising and reporting the results. The sixth step ‘consultation exercise’ being an optional component of the scoping study framework (Arksey & O'Malley, 2005), will not be included in the present review. This approach will provide a context to address the current review’s queries. Subsequently, the search strategies will be detailed before presenting the results.

### An evolutionary understanding of loneliness

For the purpose of this paper, loneliness is understood from an evolutionary perspective, as theorised by Cacioppo et al. (2006). This perspective posits that loneliness heightens survival mechanisms towards social threats to reduce the damages that can be inflicted from poor social interactions. Lonely individuals would subsequently be motivated to search for and maintain social connections to ensure survival and security. Experiencing loneliness over time can result in poor physiological and psychological health and its effects can be mitigated by interpersonal connections that function similarly to that of other basic human needs such as hunger and thirst. An experimental study by Poerio et al. (2016) reported that lonely individuals, who participated in daydreaming about socialising with a close other, showed a significant improvement in feelings of connectedness and belonging. The outcome indicates that the sense of belonging resulting from engaging in a pleasant social activity perhaps provides a rewarding, emotional benefit that alleviates feelings of loneliness.

### Loneliness and well-being

In line with the emphasis on the evolutionary perspective of loneliness, an epidemiological cohort study by Matthews et al. (2019) found that loneliness is an indicator for poor functioning in the various domains of well-being. In particular, loneliness has been reported to have strong negative associations with subjective well-being (SWB) in both cross-sectional (e.g., Chen & Feeley, 2014; Haybron et al. 2008; Hsu, 2020) and longitudinal (e.g., Shankar et al., 2015; VanderWeele et al., 2012) studies. SWB is defined as people’s cognitive and emotional perceptions of their lives (Busseri & Sadava, 2011). It reflects a positive state that drives individuals to continue maintaining this state. Conversely, as described in the previous paragraph, loneliness is understood as an aversive state that acts as a signal to change behaviour. Hence, loneliness can affect SWB through the changes in emotions and moods. To illustrate, Hsu (2020) reported that among older adults the unfulfilled expectations from the family can result in feelings of loneliness and isolation, which in turn is resulted in depressive symptoms and lower life satisfaction. Shankar et al. (2015) also noted that older individuals aged 50 years and above who experienced initial high levels of loneliness consistently have lower SWB over a six-year period. Similar outcomes are reported for young adults where evidence points to direct link between loneliness and low well-being (Fardghassemi & Joffe, 2021). These results confirm the notion that loneliness and SWB share a negative correlation. The perceptions of loneliness appear to influence an individual’s SWB despite the presence of factors that aim to promote SWB, suggesting that the aversive effects of loneliness may extend beyond negatively affecting one’s SWB to influence one’s overall life satisfaction due to the social disconnection from others since they perceive the people around them as a threat (Chernyak & Zayas, 2010).

### Social relationships and loneliness

Pitman et al. (2018) underscored the importance of understanding how the quality and quantity of social relationships among young people affect the pathway between loneliness and well-being. A qualitative meta-analysis by Achterbergh et al. (2020) noted that the experience of loneliness in later stages of life is driven by relational and environmental factors faced in childhood and adolescence. Such factors include parent-child relationships and interactions with peers. The experience of loneliness varies across sociodemographic factors. Early experiences with poor parental bonds can lead to insecure attachments with external others such as friends. This is in accordance with the attachment theory, which posits that interactions with attachment figures (e.g., parents) build an infant’s mental representation of social relationships (Bowlby, 1973). These representations subsequently influence the infant’s behaviour, thought processes, and emotions throughout the lifespan. Additionally, parent-child relationships appear to be the most influential form of social bonds. Positive interactions with parents are necessary for individuals to effectively adapt to the various developmental periods. For example, Bostik and Everall (2007) reported that insecurely attached adolescents found difficulties forming emotionally close friendships as compared to their securely attached peers. This is in line with findings from Wiseman et al. (2006) which found that undergraduate students who felt that they lacked parental care and nurturance in their childhood had impaired self-concepts. This decreased their abilities to socialise effectively, which subsequently led to a dissatisfaction in interpersonal relationships and increased feelings of loneliness. Similarly, Karababa (2021) also noted that adolescents with more positive parental attachments have higher self-esteem, which in turn led them to perceiving themselves as less lonely. The abovementioned findings imply that the quality of early bonds with parents is essential to an individual’s development, which eventually influences life satisfaction.

In younger individuals, their lives revolve around forming social networks (Bostik & Everall, 2007; Crosnoe, 2000). These social networks can act as a buffer against any ill effects of stress (Rokach, 2016). Peer rejection and decreased social support has been found significantly associated with loneliness since it might make one feel disconnected with the social reality (Heinrich & Gullone, 2006; Rokach, 2016). At a stage when young individuals are expected to form fulfilling social bonds outside of their family, deficient peer relationships may make them vulnerable to physical and psychological health issues (Chango et al., 2015; Lambert et al., 2013; Segrin & Passalacqua, 2010).

Thus, an individual’s early life experiences with social relations contribute to the experience of loneliness and well-being in later stages of life. The experience of loneliness can be minimised or exacerbated from the use of specific coping strategies. It is thus important for the mental health sector to understand what drives loneliness in young people, which prompted the current review. Keeping this as focus, the systematic scoping review was undertaken to examine the extent and range of research activity specifically focused on the experience of loneliness among young people and its subsequent effect on their well-being.

## Methodology

Utilizing the widely endorsed six-step framework described by Arksey and O'Malley (2005), the researchers charted the below mentioned steps to identify relevant works to be included in the review. As mentioned in the earlier section, we followed five of the six steps in the framework, choosing not to include the sixth stage - ‘consultation exercise’, which is viewed as an optional component of the scoping study framework (Arksey & O'Malley, 2005).

### Identifying the research question

Scoping reviews are undertaken to summarize what is known about a specific topic to date by maintaining a wide approach in order to generate breadth of coverage. (Arksey & O'Malley, 2005; Levac et al. 2009). Keeping in mind the objectives of this systematic scoping review we formed a broad research question which was: *What is known from the extant literature about the experience of loneliness among youth and its subsequent effect on their well-being?* Through this process we aimed to identify factors that could potentially influence loneliness in youth and we were also keen to know if those works focused on studying the effect of loneliness on well-being in youth.

### Identifying relevant studies

Since scoping reviews are comprehensive, the search for the relevant works was done via multiple sources. To guarantee adequate and efficient coverage, specialized and interdisciplinary electronic databases utilized in this review were APA PsycINFO, Scopus, Emerald Insight and OneSearch (Bramer et al., 2017). Grey literature, which might not be well documented or easily obtainable (Adams et al., 2016), systematic and literature reviews and intervention studies were excluded. Additional searches included checking the reference lists of all included papers. We also carried out citation searches of key publications to identify subsequent publications.

### Study selection

The team members independently searched for relevant works but the exploration of the available works yielded a large number of irrelevant studies. Discussions based on the initially reviewed articles were undertaken to draw conclusions and direct subsequent eligibility criteria. This criteria was refined as the review progressed and we learnt more about the existing body of works.

#### Eligibility criteria

This review utilised the following inclusion and exclusion criteria: in order to be included in the review, the paper had to report loneliness among youth, either as an independent variable (IV; variable that could be cause of a behaviour or an outcome), or as a dependent variable (DV; the outcome variable that depends on the IV). The review followed the definition of “youth” as individuals aged 15 to 24 years old [World Health Organisation (WHO), 2021]. Eligibility was limited to full-text, qualitative or quantitative research published within the last 10 years in a peer-reviewed English language journal. A 10-year time frame was used since boom in social technologies in the last decade have been documented to increase loneliness in modern society (Twenge et al., 2021). The ten year period would also ensure that the information extracted from the studies would have relevance in the current times (Arksey & O'Malley, 2005). Since many works on loneliness have already focused on older population (Fakoya et al., 2020) and there is a paucity of works examining the experience of loneliness among youth, the review focused on young people within the community. Papers utilising samples with pre-existing mental health issues were excluded as the researchers wanted to investigate loneliness and related factors within the general population. Though this review focused on youth, publications that included adult populations were retained if the results offered retrospective explanations on the risk factors of loneliness during the childhood or adolescence period (e.g., Stafford et al., 2016).

The review was conducted between 20 October 2020 and 30 March 2021. Based on the 10-year timeframe chosen as a criteria, initially papers published between 1 January 2011 and 28 February 2021 were included in the review. However, due to COVID-19, the research team was delayed in meeting time target for wrapping this paper. So another search for databases was done and the search date for related papers was extended to December 2021 to include more works. Search terms included Loneliness, Well-Being, Youth, Relationship, Social Support as keywords that were then combined using the Boolean operators “OR” and “AND” to merge search terms. For instance Social Support AND lonel* AND well-being; Youth OR Students AND lonel* AND well-being and; Youth OR Adolescents AND Lone*; Relationship AND lonel* AND Youth. We screened titles and abstracts to determine whether the study met the inclusion criteria for this review.

## Results

### Data extraction and collection

Database search yielded a total of 1260 papers. The journal articles were manually exported from the databases and imported into Rayyan QCRI (Ouzzani et al., 2016), a web-based application for database management of systematic reviews. After removing duplicates (*n* = 220) a total of 1040 records were left for screening.

**Fig. 1:**
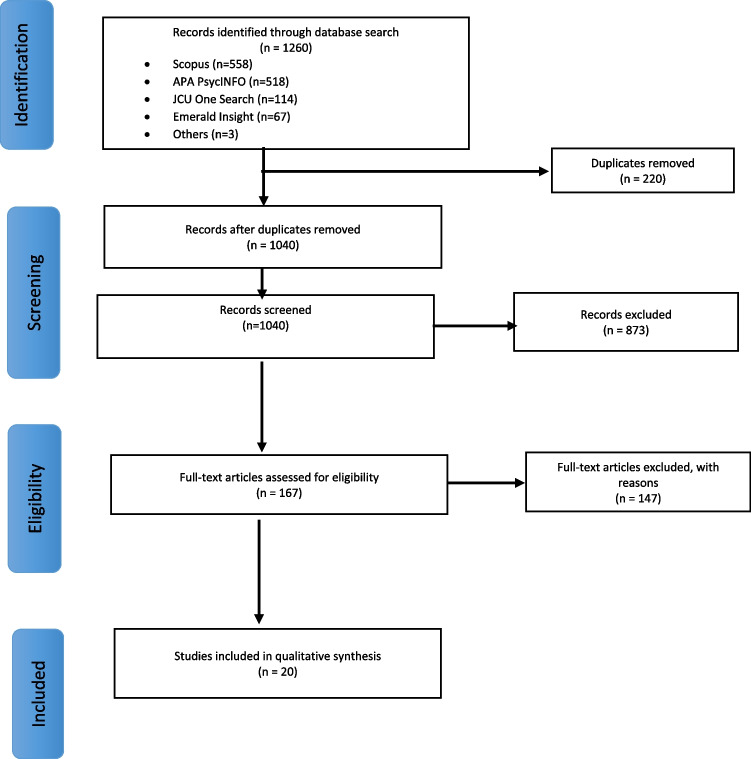
Preferred Reporting Items for Systematic Reviews and Meta-Analyses (PRISMA) flow diagram for studies examining loneliness and wellbeing among youth

Before starting the screening process, a benchmarking exercise was done to establish reliability with the blind function on. The reviewers first gave their decision on 10% of the papers and the first reviewer then turned the blind function off to reveal the decisions to the team. This allowed the team to see how many papers were unanimously agreed or disagreed upon. This initial phase of quality check when searching and selecting relevant studies is a crucial aspect of conducting a systematic review (Petrosino, 1995) since it ensures that expended time and effort is not wasted. It also standardizes the whole process and makes it more transparent and replicable (Belur et al., 2018). The benchmarking exercise showed 80% concurrence rate. The reviewers then discussed the reasons for their conflicting decisions. Following this, the inclusion and exclusion criteria were further refined and made more explicit and clearer. Once the benchmarking exercise was complete, reviewers proceeded with abstract screening. Application of the eligibility criteria identified 167 articles for full-text review. Any articles that were disagreed upon were re-read by both reviewers and discussed for relevance. Eventually, 20 papers were retained for the current review. The study selection process is summarised using a Preferred Reporting Items for Systematic Reviews and Meta-Analyses (PRISMA) flow diagram (Fig. 1).

### Chartering the data

The data from the selected articles were organised into a table in Microsoft Word, with the authors’ names arranged in alphabetical order (Table 1). The table columns comprised of author/year, country in which the research was conducted, sample demographics (age, gender), research aim, study design, measures, and relevant findings from the study.

#### Geographical location

**Fig. 2:**
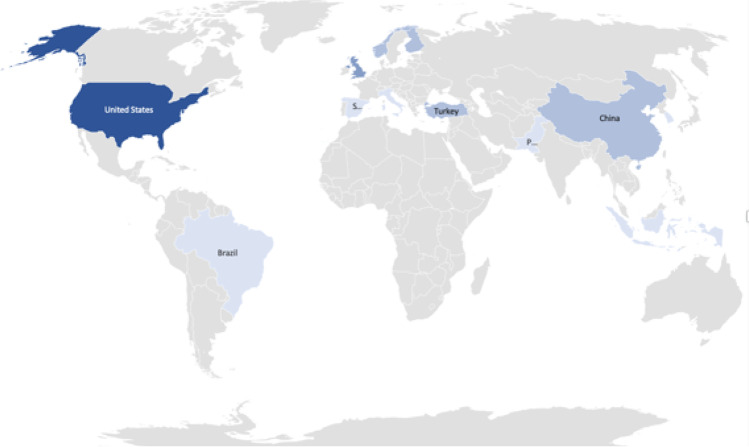
Location of the studies on loneliness among youth

The geographical range of the 20 studies included in this review was limited (Fig. 2). The studies took place in 13 out of the 195 countries listed by the Global Health Security Index (2020). Seven of the 20 studies were carried out in Europe and United Kingdom (Dalton & Cassidy, 2021; Inguglia et al., 2015; Kekkonen et al., 2020; Lyyra et al., 2021; Moksnes, et al., 2022; Phu & Gow, 2019; von Soest et al., 2020), while four studies were conducted in the United States of America (Lee & Goldstein, 2016; Newman & Sachs, 2020; Teneva & Lemay, 2020; Weisskirch, 2018). In Asia, a three studies were conducted in China (Kong & You, 2013; Tu & Zhang, 2015; Zhang et al., 2015), and one each in South Korea (Park & Lee, 2012), Pakistan (Yousaf et al., 2015) and Indonesia (Rahiem, et al., 2021). Two studies were carried out in Turkey (Arslan, 2021; Özdogan, 2021) while one study did not indicate the geographical location of participants.

#### Research designs and samples

Two major methodological patterns emerged in the included articles (Table 1- Appendix B). Firstly, majority (i.e., 14 of the 20 included studies) utilised a quantitative research design and two used a longitudinal approach (Kekkonen et al., 2020; von Soest et al., 2020). Two studies utilized qualitative approach (Janta et al., 2014; Rahiem, et al., 2021) and two made use of mixed-methods research design (Newman & Sachs, 2020; Teneva & Lemay, 2020).

Secondly, we found that 11 of the 20 studies recruited a mix of adolescent and young adult samples (Dalton & Cassidy, 2021; Inguglia et al., 2015; Kong & You, 2013; Lee & Goldstein, 2016; Moksnes et al., 2022; Özdogan, 2021; Park & Lee, 2012; Phu & Gow, 2019; von Soest et al., 2020; Weisskirch, 2018; Yousaf et al., 2015) while of the remaining nine studies, four focused on only adolescent samples (Arslan, 2021; Kekkonen et al., 2020; Lyyra et al., 2021; Zhang et al., 2015), and remaining on university students. The sample sizes for the included works ranged from 166 to 5883 participants. All studies included both males and females in their samples.

#### Tools used to measure variables

The included studies in this systematic scoping review used self-report measures of loneliness and well-being via questionnaires, interviews, diaries, focus groups and online forum threads. Studies defined the concepts of loneliness and well-being in similar ways. Loneliness was generally characterised as an unpleasant experience from the lack of quality and/or quantity of social relations. Though the authors were in agreement on the definition of loneliness, different studies chose to focus on different aspects of loneliness. Such aspects included feelings of neglect or disparities between the past and expected social inclusion. Majority of the studies (*n* = 13) utilised the University of California, Los Angeles (UCLA) Loneliness Scale or one of its versions as a measure of loneliness.

Most studies defined well-being as a multidimensional concept which included notions such as self-esteem and life satisfaction. Rosenberg-Self-Esteem-Scale was the most commonly used tool (RSES; *n* = 4; Kong & You, 2013; Park & Lee, 2012; Teneva & Lemay, 2020; Weisskirch, 2018), followed by Warwick–Edinburgh Mental Well-being Scale ( long and short versions) (WEMWBS; *n* = 3; Dalton & Cassidy, 2021; Lyyra et al., 2021; Moksnes et al., 2022), 2013), Subjective Happiness Scale (SHS; *n* = 2; Phu & Gow, 2019; Weisskirch, 2018) and Satisfaction with Life Scale (SWLS; *n* = 2; Kong & You, 2013; Tu & Zhang, 2015).

### Findings

This section consolidates themes that emerged from this review. Multiple factors such as parental bonding and relationships, personality, social support and stress emerged as significant contributors to experience of loneliness among youth, which in turn impacted their well-being.

#### Loneliness as multidimensional construct

Though most of the existing works (*n* = 16) approached loneliness as a unidimensional experience, a few studied loneliness as a multidimensional construct. Teneva & Lemay (2020) in their study examined the different impact of trait and state loneliness on self-esteem and affect. Their results showed that both trait and state loneliness could distort a person’s vision of future and lead them to expect and experience worse outcomes. von Soest et al. (2020) conceptualized loneliness as having social and emotional facets. While emotional loneliness increased across adolescents and young adulthood years, social facet of loneliness plateaued when individuals reached their mid-20 s. Özdogan (2021) examined emotional facet of loneliness further by breaking down into romantic and family loneliness. Their study found subjective well-being to be negatively correlated to romantic and family loneliness. Increase in social and emotional loneliness predicted meaninglessness of life.

#### Parental bond as a protective barrier against loneliness

Positive parental bonding was frequently reported as a protective barrier against loneliness among young people (Inguglia et al., 2015; von Soest et al., 2020; Weisskirch, 2018). Notably, these studies explicitly reported the strong influence of past memories of nurturing and loving parents on the individual’s current state of loneliness and well-being. von Soest et al. (2020) noted that perceived parental support predicted higher autonomy and relatedness in adolescents and young adults, which, in turn, predicted lower loneliness. Weisskirch (2018) too argued that young adults’ experience with parenting influenced their ability to achieve intimacy resulting in personal enhancement associated with well-being. Thus, close bonding with parents, albeit indirectly, in a way protected the young adults from negative psychological outcomes emerging out of loneliness. Young adults who perceived their parents as more supportive of their sense of relatedness reported higher levels of connectedness to others, which in turn negatively associated with loneliness (Inguglia et al., 2015). Their study highlighted the relevance of supportive parenting for psychological well-being in emerging adults.

#### Social support as an inhibitor of loneliness

Majority of the included studies gave evidence for the inhibiting effect of social support on loneliness ( Inguglia et al., 2015; Janta et al., 2014; Kekkonen et al., 2020; Kong & You, 2013; Lee & Goldstein, 2016; Park & Lee, 2012; Phu & Gow, 2019; von Soest et al., 2020; Yousaf et al., 2015; Zhang et al., 2015). These studies frequently implied that social support from family and friends was effective in decreasing the experience of loneliness. According to Inguglia et al. (2015), a sense of relatedness to other people acted as a protection against feelings of loneliness. Social support was a significant mediator between interpersonal relationship and loneliness, specifically in the school setting (Zhang et al., 2015). Not having close friends in adolescence was related with higher experience of loneliness ( von Soest et al., 2020). According to them, this could influence the future social trajectory of these individuals, where they could potentially lack friends and support network during young adulthood stage. Presence of carings realtionships resulted in sense of worthiness in adolescents and youths and reduced their need to daydream as a way of coping with loneliness (Yousaf et al., 2015). Having more friends on Facebook resulted in lower levels of loneliness and higher subjective happiness (Phu & Gow, 2019). Bonding relation and social support were strongly and negatively related to the level of loneliness in college students (Park & Lee, 2012). This was similar to findings by Yousaf et al. (2015), Kong & You, (2013), and Janta et al. (2014), who too found that perception of social support was negatively correlated with loneliness among undergarduate students, with those low in social support experiencing more loneliness. Social support as a stress buffer against loneliness varied for different sources of support ( Lee & Goldstein, 2016). For college aged youth, only support from friends and romantic partners negatively associated with loneliness. Family support was not associated with loneliness. Quality of realtionship with teachers and same sex friends too reduced the experience of loneliness in adolescents ( Zhang et al., 2015).

#### Social media, loneliness and wellbeing

Of particular interest, social media was found to influence the pathway between loneliness and well-being. However, there were mixed findings regarding its influence. For example, Park and Lee's study (2012) found that the use of smartphones for supportive communication among university students improved social bonds and psychological well-being. Janta et al. (2014) found that online social interactions could act as a form of social support. They reported that doctoral students utilised online social platforms as a means of escape from their experience with loneliness in school. Also having friends on social networking sites acted as a buffer against loneliness. Phu and Gow (2019) found that having greater number of Facebook friends positively correlated to subjective happiness and negative correlated to loneliness. However, their study found no associations between loneliness and the time spent on Facebook among young adults. These findings have implications, especially in the present times, when COVID-19 driven changes to social interactions encourages us to use social networking sites more often as compared to pre COVID-19 days. Not all time spent on such sites is fruitful and contributing to our well-being. Outcomes from the review showed that Internet and social networking sites could both be tools to forge and nurture online relationships as well as lead to risky, impulsive, or excessive usage in lonely users. Fulfilling engaging relationships not only reduce loneliness but also can help control dysfunctional online behaviours like problematic or addictive internet usage that can result in social, emotional, physical, or functional impairment (Dalton & Cassidy, 2021).

#### Loneliness, well-being and mental health

Feeling lonely can have debilitating consequences for well-being and mental health. In adolescents, where on one hand sense of belongingness acts as a buffer against emotional and behavioral problems, loneliness has adverse impact on psychosocial development. Relationships play a significant role in our life and this is true for the youth as well. Loneliness has shown a strong effect on well-being, by increasing the absence of well-being and decreasing positive well-being. Arslan (2021) found that adolescents who had low levels of inclusion at school reported greater feelings of loneliness and mental health problems. In their study, loneliness also had a negative association with subjective well-being. Similarly, being lonely was associated with lower life satisfaction in young adults as well (Kekkonen et al., 2020; Kong & You, 2013; Tu & Zhang, 2015). Loneliness was negatively related to affective well-being (Newman & Sachs, 2020) and psychological well-being (Teneva & Lemay, 2020). The COVID-19 pandemic isolation generated strong feelings of emotional distress among a sample of university undergraduates in Indonesia who reported confusion, anger, frustration and unceratinity about their future (Rahiem, et al., 2021). Loneliness was highlighted as a significant risk factor for mental wellbeing and self-esteem in Nordic adolescents as well (Lyyra et al., 2021). Reviewed works also showed that loneliness is associated with an increased risk of certain mental health problems, including depression and anxiety (Inguglia et al., 2015; Janta et al., 2014; Kekkonen et al., 2020; Moksnes et al., 2022; Park & Lee, 2012; Tu & Zhang, 2015; von Soest et al., 2020), increased stress (Inguglia et al., 2015; Lee & Goldstein, 2016), diminished self-esteem (Lyyra et al., 2021; Teneva & Lemay, 2020), and less positive affect (Newman & Sachs, 2020; Teneva & Lemay, 2020).

## Discussion

The two questions that directed this systematic scoping review brought attention to the relationship between loneliness and well-being, and how this pathway is affected by certain key factors identified in the literature. In summary, this review of 20 articles reinforces understandings of the experience of loneliness as a complex, evolutionary process that is influenced by relational and environmental factors. It further suggests that there are strong similarities between the experience of loneliness in different parts of the world. Insights can be concluded from the current review about the experience of loneliness on well-being among young people.

### The importance of relational support

It is noteworthy that the included studies reported a high number of loneliness-prevention processes at the relational level of young people. Acknowledging this fits with the argument that positive social resources, including friends and family, can play a crucial role in abating loneliness. In other words, the attempts by mental health practitioners to tackle the issue of loneliness among young people should consider the relational factors. To this end, loneliness interventions should not prioritise only relational processes over other resources (e.g., environmental), or vice versa. This could be tricky, given the fact that close relationships have been recognised as a protective factor (Chang et al., 2017; Kalaitzaki & Birtchnell, 2014; Segrin & Passalacqua, 2010). Nonetheless, tapping on the bidirectional nature of relationships, mental health practitioners could enhance the support given to young individuals and their social systems in learning ways of advancing loneliness-prevention exchanges.

It should be noted that although the relational resources were relatively similar across the included studies, applying this notion to specific contexts should be done with caution. To illustrate, significant ethnic differences were found in the relationship between parental bonding and loneliness (Weisskirch, 2018). This means that appreciation of intimacy between the parent and child may not resonate with all across cultures. The cultural orientations or ethnic differences may cause the child to prioritise other external relationships (e.g., friends) over parental relationship to deal with the loneliness. Thus, mental health practitioners should caution against the assumption that a specific relational resource is ubiquitously protective in all young people. Instead, they should target the protective relationship that is relevant to that particular young individual. In doing so, practitioners enhance collaboration between them and their clients in the therapeutic process, and increase their chance at success by exploring acceptable means of moderating the challenges that a specific context imposes on the individual.

### Impact of loneliness on well-being and mental health of youth

This scoping review identified the adverse effect of loneliness on emotional health of young population. Loneliness resulted in not only poor mental health but also significantly lowered positive well-being among youth. Experiences of diminished life satisfaction, lower affective and psychological well-being, among lonely youth, thus necessitates more focused research on loneliness experiences among the young and adolescent populations. Reduced or limited access to friendship groups or peers have a psychological cost for youth as many participants in the included works showed symptoms of poor mental health including anxiety and depression. With world now emerging from the shadows of CoVID-19 pandemic, there is an urgent need to focus on the emotional well-being of the young population. The debilitating effects of loneliness among youths have to be acknowledged by the society in order to start conversations on this not-so-trivial issue.

### Contributions of social media in current times

Apart from the importance of relational support for managing loneliness among youth, this review also identified the contributions of social media as a tool for social support. The fact that the loneliness of young people was intertwined with social media usage could be due to globalisation and boom in social technologies. The use of social networking sites was means of escape from reality (Janta et al., 2014) but this was also linked to a reinstated sense of happiness and belonging (Phu & Gow, 2019).

Despite the contributions of social media usage in decreasing loneliness, individuals may not reap equivalent benefits at par with face-to-face social support or interactions. Significantly, one study (i.e., Kekkonen et al., 2020) emphasised the importance of in-person social interactions. Kekkonen et al. (2020) noted that lower frequencies of physically meeting friends significantly decreased life satisfaction among young adults. These findings gain significance in light of the social distancing measures in place across many countries to prevent the spread of CoVID-19. In such circumstances, social media could offer a safe alternative and be a valuable platform for lonely individuals seeking social support and interactions. In present times when COVID-19 driven restrictions might force people to limit their face-to-face interactions, social media could be an effective tool to manage the consequences of loneliness.

What stood out in the review was limited works examining the experience of loneliness in youths. The body of reviewed works showed that the youth too are vulnerable to experiencing loneliness and suffer its consequences. Some parts of the world are still focused on fighting COVID -19 by imposing restrictions on social gatherings and limiting social contact if need be. Since youths thrive on social interactions and engagements, the impact of these restrictions on their emotional health might be more severe than other demographics within the society. Youth are our future and we need to now focus on their experience of loneliness. Studies focused on examining and understanding their experiences are required so that solutions can be offered to mitigate the effect of loneliness in order to ensure that this valuable demographic does not suffer silently.

### Limitations

This review is not without limitations. Firstly, logistical constraints limited the ability to consult with relevant stakeholders (e.g., loneliness-focused practitioners). Given the vast research conducted on loneliness and well-being, as well as the dynamic nature of social media (Phu & Gow, 2019) and parent-child relationships (Lunkenheimer et al., 2017), this review will require an update. Ideally, it should be done with the consultation of relevant stakeholders. Secondly, the search for articles excluded non-English language journals. This creates the possibility that the current review could have reduced relevant insights on the topic of interest in other languages. Thirdly, the methodology did not focus on quality assessment of the included research papers. This is reflective of the scoping review approach (Arksey & O'Malley, 2005). Nonetheless, quality assessment should be considered in the future, especially if the findings from this review are going to help direct future interventions. Lastly, this review only focused on empirical works and did not include literature reviews, systematic reviews, interventions and grey literature. Due to not including all available data on this topic, a selection bias might have occurred. Since for this review only four databases were utilised, with searched articles limited to the last 10 years, these criteria may have limited the scope of findings.

## Conclusion

There is a rising public health concern worldwide due to the social distancing measures that were brought in to mitigate the effect of COVID-19 pandemic. In this systematic scoping review, information was gathered to shed light on the current evidence related to the experience of loneliness in young people and how this affects their well-being. Essentially, their capacity to ameliorate loneliness is rooted in their early relationships with parents, their social support and the ways in which they choose to cope with the loneliness (e.g., using social media to connect with others). Understanding that these factors feature in empirical accounts of adolescents’ and young adults’ loneliness is vital to the mental healthcare sector’s efforts to tackle this problem. Emerging adulthood is an important transition period with focus on growth, gains, and planning for positive future. However, COVID-19-related stressors and disruptions led to social isolation, confinement, loss of freedom, and a sense of uncertainty that significantly impacted the mental health and psychosocial well-being of youth. This systematic scoping review did not find many works that focused on the experience of loneliness among youth in community. Studies that measure loneliness and well-being at different time points appear to be need of the hour. This will allow researchers to assess the temporal unfolding of loneliness at different intensities of the imposed social distancing measures. Studies need to be replicated and utilise more rigorous designs to validate the results from the included works in this review. By addressing these factors, researchers can help facilitate better mental health in the young population.

## Data Availability

The datasets generated during and/or analysed during the current review are available from the corresponding author on reasonable request.

## Appendix A

Figure 1

Figure 2

## Appendix B

Table 1

**Table 1:** Characteristic description of studies (*n* = 20) included in the systematic scoping review

Author & Year	Country	Sample	Design	Purpose	Measures *	Outcomes
1. Arslan, 2021	Turkey	244 adolescents aged 14–18 years	Paper pencil survey	To investigate the mediating effect of loneliness on the association between school belonging constructs (i.e., social inclusion and exclusion) and subjective well-being and mental health problems	SBS, YIBS & YEBS, UCLA-8, SWLS	School-based social inclusion and exclusion significantly predicted loneliness, mental health problems, and subjective well-being. Loneliness partially mediated the relationship of social inclusion with mental health problems and subjective well-being
2. Dalton & Cassidy, 2021	UK	673 emerging adults aged between 18–25 years	Cross-sectional online survey	Explore the relationship between problematic Internet use, the big five dimensions of personality, loneliness, and well-being in a sample of emerging adults	OCS, UCLA-R,TIPI, WEMWBS	Problematic Internet use had a highly significant inverse relationship with well-being, and a small but significant direct relationship with loneliness
3. Inguglia et al., 2015	Italy	325 Caucasian adolescents and emerging adults aged 17–26 years old	Cross-sectional survey	(a) Examine autonomy and relatedness in late adolescents & emerging adults (b) Analyse the relationships with perceived parental support and psychological distress	BPNS, PPS, YSR, ASR, UCLA, DASS-21	Autonomy and relatedness negatively predicted loneliness. The development of a sense of relatedness to other people appeared to protect individuals from negative emotional states related to depression, feelings of loneliness, and stress during adolescence and emerging adulthood. Parental support positively predicted autonomy and relatedness
4. Janta et al., 2014	Not specified	Doctoral students	Netnographic study	Understand how doctoral students cope with loneliness and isolation, and what tactics are used to overcome such issues	Active sites for doctoral students	Both domestic and international students from a range of disciplines experienced social isolation, suffered a lack of emotional support and struggled to engage in meaningful relationships with their peers. Loneliness occurred at different stages of the doctoral study. Participants used tactics including multiple forms of (face to face and online) social interaction, professional development and escape from the doctorate to deal with loneliness
5. Kekkonen et al., 2020	Finland	787 adolescents aged 13–18 years old	Longitudinal – 5-year follow-up study	Examine the associations between social activities, relationships, and alcohol consumption of adolescents and their life satisfaction until adulthood	YSR of ASEBA, BDI-21, AUDIT-C, LS-4	Experience of loneliness and lower frequency of meeting friends and in adolescence linked with lower life satisfaction in young adulthood, especially in males. Loneliness mediated the effect of the frequency of meeting friends in adolescence on life satisfaction in young adulthood Frequently meeting friends was related to lower loneliness, which in turn was linked to lower BDI scores
6. Kong & You, 2013	China	389 Chinese college students aged 17–25 years old	Cross-sectional survey	Examine the mediation effects of loneliness and self-esteem on the relationship between social support and life satisfaction	SWLS, SELS, CSSRS, RSES	Individuals with low levels of social support were likely to engage in loneliness and have low self-esteem, which could result in low life satisfaction. Self-esteem and loneliness, fully mediated the relationship between social support and life satisfaction
7. Lee & Goldstein, 2016	USA	636 ethnically diverse university students aged 18–25 years old	Cross-sectional survey	Examine the stress-buffering function of social support against loneliness Determine the role of gender in these associations	PSS-10, MSPSS, UCLA-8	Support from friends buffered the association between stress and loneliness. Also, support from friends (not family) was negatively associated with loneliness. Gender moderated the association between social support and loneliness
8. Lyyra et al., 2021	Denmark, Finland, Iceland, and Sweden	5883 15-year-old boys and girls	Cross-sectional survey- paper and pen (Sweden) electronically (Finland, Denmark, and Iceland)	Compare the prevalence of loneliness, mental well-being, and high self-esteem across the four Nordic countries and to examine how loneliness is associated with mental well-being and self-esteem	Single-item measure of loneliness, SWEMWBS, 3-item self-developed measure of self esteem	Loneliness was a significant risk factor for participants’ mental well-being and self-esteem in all four countries. Feelings of loneliness were most frequent in Finland with 19% reporting loneliness and the lowest prevalence was in Denmark (8%). Girls reported higher rates of loneliness compared to boys in all four Nordic countries
9. Moksnes et al., 2022**	Norway	1816 adolescents aged 15–21 years	Teacher administered survey	Investigated sociodemographic differences (sex, age, self-reported socio-economic status (SES)) in loneliness and associations between sociodemographic factors, loneliness and self-rated health, subjective health symptoms, symptoms of depression/anxiety and mental well-being	SWEMWBS, HSCL-10,SRH ( one item), 11 items SHC, Single-item measure of loneliness, single item measure of perceived relations	Significant sex differences in perceived loneliness and the health outcome variables investigated. Also significant association between higher level of loneliness and symptoms of depression/anxiety was reported
10. Newman & Sachs, 2020	USA	504 university students (age range not specified) with mean age of 20.10 years old	Mixed-methods (survey and qualitative analysis of diary logs)	Examine if the effect of nostalgia on well-being depends on the event or experience that elicits nostalgia	4-item PINE, affective circumplex model	The negative effects of nostalgia on affective wellbeing were significantly stronger on days when people felt more lonely as opposed to less lonely. Loneliness and nostalgia were negatively related to affective well-being
11. Özdogan, 2021	Turkey	477 university students aged between 18–33 years	Cross-sectional survey ( mode not specified)	Investigated the relationships between social and emotional loneliness, the meaning and purpose of life and subjective well-being, and to find out direct and indirect predictors of these relationships	PANAS, MPLS, SELS	Social and emotional loneliness significantly predicted the meaning and meaninglessness of life, and the meaning and meaninglessness of life significantly predicted subjective well-being
12. Park & Lee, 2012	South Korea	270 university students aged 18–28 years old	Cross-sectional online survey	Explore the relationship between motives of smartphone use, social relation, and psychological well-being	Self-report 5-point and 7-point Likert scales, MSPSS, RSES, R-UCLA, CES-D (7-item version)	Individuals who used smartphones to fulfill their caring motive were likely to show lower levels of loneliness and depression and maintain greater self-esteem. A smartphone could serve as a platform through which students can socialize with others, thus contributing to improving emotional and psychological well-being
13. Phu & Gow, 2019	UK	332 participants aged 18–29 years old	Cross-sectional online survey	Determine the aspects of Facebook usage linked to subjective happiness and loneliness	MFIS, SHS, UCLA	Loneliness emerged as a significant negative predictor of subjective happiness. Persistent usage of Facebook, was associated with higher levels of loneliness. Higher number of Facebook friends predicted subjective happiness and lower levels of loneliness. However, time spent on Facebook did not significantly predict loneliness. No gender differences in subjective happiness and loneliness were observed
14. Rahiem, et al., 2021	Indonesia	166 university students	Qualitative	The study explored how youths perceived their well-being after being isolated for one-and-a-half years during the COVID-19 pandemic	Essay question utilizing narrative inquiry approach	Seven themes emerged describing how the students perceived their well-being after 1.5 years of being isolated during the COVID-19 pandemic: (1) the anguish of loneliness and estrangement; (2) a state of “brokenness”—emotional agony and distress; (3) frustration, confusion, and anger; (4) the experience of conflicting emotions;(5) uncertainty about current and future events; (6) a sense of purpose and fulfillment; and (7) turning to faith
15. Teneva & Lemay, 2020	USA	187 college students	Mixed-methods (survey and qualitative analysis of diary logs)	Assess the effects of loneliness on self-esteem and affect	RSES, PANAS, UCLA LS-III, UCLA-8, self-report 7-point Likert scales, self-report 5-point Likert scale	People who were chronically lonely were likely to recall being excluded in the general past and expected to be excluded in the general future. Daily feelings of loneliness also indirectly predicted reduced daily self-esteem and positive affect
16. Tu & Zhang, 2015	China	444 undergraduates	Cross-sectional paper-pen survey	To examine the effects of loneliness on individuals’ stress, depression, and life satisfaction and role of self- efficacy as a mediator	UCLA-R, GSE, PSS-14,CES-D,SWLS	Loneliness was a significant predictor of high stress and depression, and low self-efficacy and life satisfaction. Self-efficacy mediated the relationship between loneliness and stress, depression, and life satisfaction
17. von Soest et al., 2020	Norway	3,116 Norwegians aged 13 to 31 years old	Longitudinal – 4-wave study over a 13-year period	Explore how loneliness develops during adolescence and young adulthood	Self-report 4-point Likert scale, UCLA (4-item version), PBI (5-item version), BSRI (short form), national register data	Loneliness was found to increase across the adolescent and young adulthood years, whereas social facets of loneliness decreased and plateaued when people had reached their mid-20 s Perceiving one’s parents as caring, having close friends and reporting more agency were each associated with reduced loneliness. Social loneliness and age were negatively correlated. Low levels of parental care were consistently associated with higher levels on all three measures of loneliness used in the study. Women reported more loneliness at all ages. Adolescents and young adults who reported feeling lonely and/or increase in loneliness were consistently at higher risk for disability and lower income in midlife
18. Weisskirch, 2018	USA	232 college students aged 18–25 years old	Cross-sectional online survey	Understand how individuals’ sense of intimacy develop its subsequent effect on depressive symptoms, loneliness, self-esteem, and happiness	EPSI, ECR-RS, PBI, PCQ-10, SERR, CES-D; UCLA LS-III, SHS, RSES	Emerging adults’ perceptions of their parenting may relate to their achievement of intimacy. Achieving intimacy, especially during emerging adulthood, may be associated with wellbeing. Intimacy was positively associated with parental care, happiness and self-esteem. Significant effect of intimacy on well-being was reported. Those individuals with low intimacy were higher on loneliness, lower on self-esteem, and lower on happiness than those with high intimacy
19. Yousaf et al., 2015	Pakistan	177 college students aged 19–35 years old	Cross-sectional survey	Examine the relationship among daydreaming, loneliness and perceived social support	Urdu-translated: SIPI, UCLA, ISEL	Perceived social support was a significant negative predictor of loneliness No significant gender differences were found in loneliness and perceived social support
20. Zhang et al., 2015	China	1674 Chinese adolescents aged 13–19 years old	Cross-sectional survey	Explore the associations between the qualities of different types of relationships in school, social support and loneliness in adolescence	UCLA, MSPSS, IRS	Same-sex relationships showed strong association with adolescents’ loneliness, indicating these relationships play an important role in adolescents’ loneliness. Boys’ loneliness was influenced by the qualities of opposite-sex, teacher–student and same-sex relationships. Girls’ loneliness was only influenced by same-sex relationships. Social support mediated the association between same-sex relationships and teacher–student relationships, and loneliness

* ASR, Achenbach family of instruments: Adult Self-Report; AUDIT-C, Alcohol Use Disorders Identification Test (consumption); BDI-21, 21-item Beck Depression Inventory; BPNS, Basic Psychological Needs Scale; BSRI (short form), Bem Sex-Role Inventory; CES-D, Center for Epidemiological Studies Depression Scale; CSSRS, Chinese Social Support Rating Scale; DASS-21, Depression Anxiety Stress Scale-21; ECR-RS, Experiences in Close Relationships-Relationships Structures Questionnaire; EPSI, Erikson Psychosocial State Inventory; GSE, General Self-Efficacy scale; HSCL-10, 10-item version of the Hopkins Symptom Checklist; IRS, Interpersonal Relationship Scale; ISEL, Interpersonal Support Evaluation List; LS-4, four-item Life Satisfaction; MFIS, Multidimensional Facebook Intensity Scale; MPLS, Meaning and Purpose of Life Scale; MSPSS, Multidimensional Scale of Perceived Social Support; OCS, Online cognition Scale; PANAS, Positive and Negative Affect Scale; PBI, Parental Bonding Instrument; PBI-5, five-item Care subscale of a revised version of the Parental Bonding Instrument; PCQ-10, Parental Challenge Questionnaire; PINE-4, Personal Inventory of Nostalgic Experiences; PPS, Perceptions of Parents Scale; PSS-10, 10-item shorter version of the Perceived Stress Scale; PSS-14, 14 item Perceived Stress Scale; RSES, Rosenberg Self-Esteem Scale; SBS, School Belongingness Scale; SELS, Social and Emotional Loneliness Scale; SERR, Self-efficacy in Romantic Relationships Scale; SHS, Subjective Happiness Scale; SHC, Subjective Health Complaints; SIPI, Short Imaginal Processes Inventory; SRH, Self-Rated Health; SWLS, Satisfaction with Life Scale; TIPI, 10-item Personality Inventory; UCLA LS-III, UCLA Loneliness Scale (Version 3); UCLA, UCLA Loneliness Scale; UCLA-8, 8-item short-form of the UCLA Loneliness Scale; UCLA-R, revised UCLA Loneliness Scale; SWEMWBS,Warwick-Edinburgh Mental Well-being Scale ( short version); WEMWBS, Warwick–Edinburgh Mental Well-being Scale; YIBS & YEBS, Youth Internalizing and Externalizing Behavior Screeners; YSR, Achenbach family of instruments: the Youth Self-Report.

** Available in 2021
